# Clinical significance of the induction of macrophage differentiation by the costimulatory molecule B7-H3 in human non-small cell lung cancer

**DOI:** 10.3892/ol.2014.2056

**Published:** 2014-04-08

JING SUN, YONG MAO, YANG-QIN ZHANG, YUN-DI GUO, CHUAN-YONG MU, FENG-QING FU and XUE-GUANG ZHANG

Onc Lett 6: 1253-1260, 2013; DOI: 10.3892/ol.2013.1586

After the publication of the article, the authors noted that they had made some errors regarding certain data in their manuscript.

We hereby retract our paper ‘Clinical significance of the induction of macrophage differentiation by the costimulatory molecule B7-h3 in human non-small cell lung cancer’ published in Oncology Letters on November. Following further research based on this article, some ambiguous results were obtained, and consequently more results needed further confirmation. We are currently working on well-designed experiments to get more conclusions. We apologize for any inconvenience that may have resulted from the publication of this paper.

